# Correction for Gurda et al., “Mapping a Neutralizing Epitope onto the Capsid of Adeno-Associated Virus Serotype 8”

**DOI:** 10.1128/jvi.00792-25

**Published:** 2025-06-12

**Authors:** Brittney L. Gurda, Christina Raupp, Ruth Popa-Wagner, Matthias Naumer, Norman H. Olson, Robert Ng, Robert McKenna, Timothy S. Baker, Jürgen A. Kleinschmidt, Mavis Agbandje-McKenna

## AUTHOR CORRECTION

Volume 86, no. 15, p. 7739-7751, 2012, https://doi.org/10.1128/jvi.00218-12. Page 7748, Fig. 7: The middle panel of Fig. 7A, which depicts the rAAV2 dot blots, appears to show a duplicated image for the trypsin-treated (– and +) samples. Because of the long passage of time since this work was published, we no longer have access to the original records. It is very possible that the rAAV2 dot blots were unintentionally duplicated during the assembly of Fig. 7, particularly as the original images were most likely visually similar.

Despite this apparent error, we believe the overall conclusion drawn from Fig. 7 remains valid. Figures 7B and C present the numerical quantification of the dot blot data and do show subtle but discernible differences between the – and +trypsin treatments. This suggests that the original blot data varied and the error likely occurred during figure preparation rather than experimentation. This pattern of slight variation is also observed in the top and bottom panels of Fig. 7A (rAAV8 and rAAV2-RGNRQ/QQNTA), and these trends are consistently reflected in the accompanying quantitative analysis (Fig. 7B and C).

In conclusion, while we agree that the rAAV2 dot blot image (Fig. 7A, middle panel) is flawed, we do not believe this affects the central findings or conclusions of the study. Nonetheless, we appreciate the thorough review that led to the finding and investigation of this error.

